# Threshold Effects of PM_2.5_ Exposure on Particle-Related Mortality in China

**DOI:** 10.3390/ijerph16193549

**Published:** 2019-09-22

**Authors:** Bao-Linh Tran, Ching-Cheng Chang, Chia-Sheng Hsu, Chi-Chung Chen, Wei-Chun Tseng, Shih-Hsun Hsu

**Affiliations:** 1 Department of Applied Economics, National Chung Hsing University, Taichung 402, Taiwan; 2 Institute of Economics, Academia Sinica, Taipei 11529, Taiwan; 3 Department of Agricultural Economics, National Taiwan University, Taipei 10617, Taiwan

**Keywords:** air pollution, energy consumption, population-weighted PM_2.5_ exposure, cardiovascular mortality, respiratory mortality, panel threshold model

## Abstract

Ambient air pollution from energy use and other sources is a major environmental risk factor in the incidence and progression of serious diseases, such as cardiovascular and respiratory diseases. This study elucidates the health effects of energy consumption from air pollution in China based on multiple threshold effects of the population-weighted exposure to PM_2.5_ (fine particles less than 2.5 microns in diameter) on particle-related mortality rate. We firstly estimate the causal relationship between coal consumption and PM_2.5_ in China for 2004–2010 using a panel regression model. Panel threshold models are applied to access the non-linear relationships between PM_2.5_ and cause-specific mortality rates that indicate the health effects are dependent on the PM_2.5_ ranges. By combining these steps, we calculate the health impacts of coal consumption based on threshold effects of PM_2.5_. We find that a 1% coal consumption increase induces a 0.23% increase in PM_2.5_. A triple threshold effect is found between PM_2.5_ and cardiovascular mortality; for example, increasing PM_2.5_ exposure causes cardiovascular mortality rate to increase when PM_2.5_ lies in 17.7–21.6 μg/m^3^ and 21.6–34.3 μg/m^3^, with the estimated increments being 0.81% and 0.26%, respectively, corresponding to 1% PM_2.5_ increase. A single threshold effect of SO_2_ on respiratory mortality rate is identified and allows the estimation of the mortality effects of PM_2.5_ regarding the two regimes of SO_2_. Finally, we access the health impacts of coal consumption under specific estimated thresholds. This study provides a better understanding of sources contributing to related-air pollution mortality. The multi-threshold effect of PM_2.5_ could be considered for further applications in harmonizing emission standards in China and other developing countries.

## 1. Introduction

${\mathrm{PM}}_{2.5}$ is derived predominantly from principal air pollution that is caused directly by gases like nitrogen oxides (${\mathrm{NO}}_{x}$), sulfur dioxide (${\mathrm{SO}}_{2}$), and carbon monoxide (CO) from fuel combustion emission in power generation, manufacturing, transportation, etc. Since ${\mathrm{PM}}_{2.5}$ could infiltrate deeply into the gas-exchange region of the lungs, and smaller particles can cross alveolar membrane into the blood vessels [1,2,3], it is the most health-damaging particle, and tends to be associated with the mortality risks from cardiovascular disease [4,5,6,7,8] and respiratory system diseases [9,10,11,12,13].

In China, known to be the largest developing country in the world, a heavy reliance on coal as a cheap and major source of energy for maintaining essential industrial activities, for instance, operating coal-fired power plants, is considered to be the main source of gaseous air pollutants, including ${\mathrm{PM}}_{2.5}$. Such a strong causal relationship between coal burning and increasing ${\mathrm{PM}}_{2.5}$ in China has been proved in previous studies [14,15,16]. As a result, air quality degradation in China related to high levels of ${\mathrm{PM}}_{2.5}$ has put huge pressures on the environment and public health. According to the National Bureau of Statistics of China, cardiovascular disease and respiratory disease ranked among the top five causes of death at the national level from 2000–2015. Because of the seriousness of and popular interest in air pollution and mortality, accumulating studies have investigated ${\mathrm{PM}}_{2.5}$ and found significant impacts on fatal damage to the cardiovascular system and respiratory disease [17,18,19]. As can be seen, there is a transitive relationship between coal consumption, ${\mathrm{PM}}_{2.5}$ and human mortality that could be studied further and more integratedly. In other words, instead of directly studying the association between consumption of coal and health consequences, the impacts of energy consumption on mortality rates could be clarified through the health effects of ${\mathrm{PM}}_{2.5}$ to gain a better understanding of the sources contributing to air pollution and polluted air-related mortality.

Regarding the determination of the relationship between mortality and fine particulate matter, many researchers initially focused on the shape of the concentration-response curve to reveal that the relationship between mortality and exposure to ${\mathrm{PM}}_{2.5}$ is nonlinear and speculated that one or more ${\mathrm{PM}}_{2.5}$ segments (thresholds) might exist. This has inspired epidemiologic studies to model the health effects of fine particulate matter using a variety of methods supporting nonlinearities. However, prior findings remain inconsistent: some studies have indicated that the concentration-response curve is close to linear [20,21,22], while others found evidence of nonlinearity [23,24,25], and there is still no clear evidence that supports the existence of ${\mathrm{PM}}_{2.5}$ thresholds, to our knowledge.

The traditional approach on the shapes of concentration-response (C-R) determines ${\mathrm{PM}}_{2.5}$ threshold levels exogenously, which may create some problems, such as being unable to obtain confidence intervals for threshold, and the estimates may be sensitive to the chosen threshold level [26,27] or ignoring exposure estimation errors while estimating average exposure concentration led to low-dose nonlinearities or thresholds being obscured [28]. This paper employs Hansen [26] threshold model to estimate the relationship between ${\mathrm{PM}}_{2.5}$ and particle-related mortality in China on a national scale. Specifically, we use panel threshold models to test whether there are threshold effects between ${\mathrm{PM}}_{2.5}$ and mortality, search for two or more regimes endogenously, and then estimate the effect of different pollutant regimes on mortality.

In light of the importance of identifying threshold effects of ${\mathrm{PM}}_{2.5}$ on human health, and inferring the health impacts of coal consumption from air pollution based on ${\mathrm{PM}}_{2.5}$ thresholds, the specific objectives of this research are, firstly, (1) to estimate ${\mathrm{PM}}_{2.5}$ caused by energy consumption in China; then (2) to elucidate how exposure to ${\mathrm{PM}}_{2.5}$ influences cause-specific mortality rate with threshold effects; and, finally, (3) to explore the relationship between energy consumption and mortality rate through ${\mathrm{PM}}_{2.5}$ health impacts.

To fulfil research objectives, this paper is constructed as follows. We firstly introduce the research background and review the literature. In the next section, we discuss panel data models for examining the impact of coal consumption on ${\mathrm{PM}}_{2.5}$ and panel threshold regression models for estimating the effects of ${\mathrm{PM}}_{2.5}$ on mortality rate. Then, we discuss the data set. Finally, the empirical results of each model and two-stage approach are interpreted, and some concluding remarks are presented.

## 2. Research Background

### 2.1. Energy Consumption and $PM_{2.5}$

As a basic concern about air quality in China, high concentrations of ${\mathrm{PM}}_{2.5}$ are believed to be closely related to coal consumption, which is the primary source of energy in the country. During 2000–2015, alongside the massive industrialization and urbanization, China consumed about 2.1 billion tons of coal, of more than 3 billion tons of SCE (standard coal equivalent) of the total energy consumption annually, which accounts for 70%. Among economic sectors, the coal-consuming industries such as coal-fired power plants (50% of consumed coal), cement, iron and steel, building, and coal conversion have consumed 85.8% of the total coal [29]. Coal-burning is considered the largest contributor to ambient ${\mathrm{PM}}_{2.5}$, since it negatively contributes about 40% to the ${\mathrm{PM}}_{2.5}$ population exposure in China [30]. Many papers have provided clear evidence to support the causal relationship between coal combustion and ${\mathrm{PM}}_{2.5}$ [14,15,16]. In addition to coal-burning-related causes, consumption of other fossil fuels and its products such as diesel oil, or gasoline also significantly contribute to ambient ${\mathrm{PM}}_{2.5}$ [31,32,33]. Based on these studies, energy consumption, especially for coal, gasoline, and diesel oil, are the major factors affecting the concentration of ${\mathrm{PM}}_{2.5}$ in China.

Other factors contributing to elevated levels of ${\mathrm{PM}}_{2.5}$ are also determined. For example, since unpaved road dust emissions also significantly contribute to particulate matter concentrations [34,35], we add the data of per capita area of paved road and expect a negative sign on its coefficient. Meteorological factors including temperature, precipitation, and humidity are also used as the controlling variables, because significant correlations between these climatic factors and ${\mathrm{PM}}_{2.5}$ level have been found previously [36,37,38]. In addition, since China was elected as the host country for the 2008 Summer Olympic Games, Chinese officials imposed many stringent emission limits on vehicles, industry, construction activities, and on fuel consumption in the most air-polluted regions. It is believed that there has been a major change in air quality in China, and therefore, we use a dummy variable of time (before and after 2008) to clarify this.

### 2.2. $PM_{2.5}$ and Mortality

Many studies have been published asserting significant connections between ${\mathrm{PM}}_{2.5}$ and human health with respect to short- and long-term effects in large Chinese cities. Specifically, for estimating air pollution short-term effects, Kan, London, Chen, Zhang, Song, Zhao, Jiang and Chen [19] indicated that in Shanghai, an increase of 10 $\mu g/m^{3}$ in the 2-day moving average concentration of ${\mathrm{PM}}_{2.5}\;$ corresponded to 0.36%, 0.41%, and 0.95% increase of total, cardiovascular, and respiratory mortality. For Shenyang city, Ma, Chen, Pan, Xu, Song, Chen and Kan [18] estimated that the risk of mortality of all-causes, cardiovascular, and respiratory increased by 0.49%, 0.53%, and 0.97%, respectively, in response to a 10 $\mu g/m^{3}$ ${\mathrm{PM}}_{2.5}\;$ increase. Another study conducted by Yang, Peng, Huang, Chen, Xu, Chen and Kan [17] provided more evidence that supports for short-term health effect of air pollution by showing that a 10 $\mu g/m^{3}\;$ increase in ${\mathrm{PM}}_{2.5}$ causes a 1.22% (95% CI: 0.63, 1.68) and 0.97% (95% CI: 0.16, 1.79) in cardiovascular and respiratory mortality in Guangzhou.

Due to the lack of ${\mathrm{PM}}_{2.5}$ data for the period prior to 2013, the ability to investigate the long-term effect of ${\mathrm{PM}}_{2.5}$ on human health is limited in China. Thus, the health impacts of ${\mathrm{PM}}_{10}$ pollution have been studied instead. For instance, retrospective cohort studies investigated by Zhang, et al. [39] and Dong, et al. [40] found that in Shenyang, China, a 10 $\mu g/m^{3}$ increase in ${\;\mathrm{PM}}_{10}$ leads to an increase of 67% in deaths caused by respiratory disease and a 55% increase in cardiovascular mortality. Regarding other countries, the long-term health effects of ${\mathrm{PM}}_{2.5}$ have also been widely studied, such as in the US, Canada, Netherlands, etc.

In addition to PM-related causes, mortality related to cardiovascular and respiratory diseases is also associated with increased concentrations of nitrogen dioxide (${\mathrm{NO}}_{2}$) and sulfur dioxide (${\mathrm{SO}}_{2}$). These associations have also been widely investigated by previous studies [41,42,43,44,45,46].

### 2.3. The Relationship between $PM_{2.5}$ and Mortality Rate

Back to empirical studies from the literature, a class of nonlinear exposure-response models has been applied to access the concentration-response relationship. Schwartz, Laden and Zanobetti [22] developed smooth functions using data of ${\mathrm{PM}}_{2.5}$ and daily deaths for six US cities and showed that the least-square fit of a linear association and no sign of a threshold. This finding is consistent with a previous result, applying a different methodology with ${\mathrm{PM}}_{10}$, Daniels, Dominici, Samet and Zeger [20] developed spline and threshold exposure-response models using daily time-series data for the 20 largest US cities and found that the association appeared to be linear. A similar approach was employed by Samoli, Analitis, Touloumi, Schwartz, Anderson, Sunyer, Bisanti, Zmirou, Vonk and Pekkanen [21] to estimate the relationship between ambient particles and daily mortality in 22 European cities, with the results indicating that the spline curves were roughly linear, but also suggesting that a threshold model would be reasonable for respiratory mortality cases.

Even though many studies have reported a linear relationship without threshold when modeling the concentration-response curve, accumulating studies have still made an effort to identify nonlinearity relations between fine particles and mortality with a variety of methodologies applied. Krewski, Jerrett, Burnett, Ma, Hughes, Shi, Turner, Pope III, Thurston and Calle [23] and Crouse, Peters, van Donkelaar, Goldberg, Villeneuve, Brion, Khan, Atari, Jerrett and Pope III [24] used the logarithm of fine particulate matter in the Cox survival models and showed that the log models were a better predictor of ${\mathrm{PM}}_{2.5}$-related mortality. Using a meta-regression approach, Burnett, Pope III, Ezzati, Olives, Lim, Mehta, Shin, Singh, Hubbell and Brauer [25] suggested fitting an integrated exposure-response (IER) model by incorporating information on risk from other sources of ${\mathrm{PM}}_{2.5}$ to demonstrate that the ${\mathrm{PM}}_{2.5}$-mortality association is nonlinear and more complex than assessments from concentration in logarithm form. Apte, et al. [47] also applied the IER model to access how mortality from ${\mathrm{PM}}_{2.5}$ could be reduced in response to improvements in air quality; the global concentration-mortality relationships were found to be nonlinear, especially for mortality of stroke and ischemic heart disease. In addition, Yu and Chien [13] used a spatiotemporal structured additive regression model to examine the concentration-response (C-R) relation between respiratory visits and ${\mathrm{PM}}_{2.5}$. The results emphasized a non-linearity of the respiratory health effects of ${\mathrm{PM}}_{2.5}$ on humans.

As a result, there is still no consensus on the shape of the concentration-mortality relationship, and no clear evidence supports the existence of ${\mathrm{PM}}_{2.5}$ thresholds to our knowledge. A recent study by Cox [28] has re-examined the shapes of C-R for ${\mathrm{PM}}_{2.5}$ with well-defined response thresholds and concluded that ignoring exposure estimation errors while estimating average exposure concentration has led to low-dose nonlinearities or thresholds being obscured. More appropriate approaches are required in modeling the association. Hansen [26] suggested a threshold regression technique for panel data model to test for threshold effects and to search for two or more regimes endogenously. In this study, we examine the health impacts of air pollution in China on a national scale by estimating the association between cause-specific mortalities for cardiovascular and respiratory diseases and annual average population-weighted exposure to ${\mathrm{PM}}_{2.5}$ using Panel Threshold Models as an econometric approach.

The major purpose here is to directly access the statistical significance of the multi-threshold effect of ${\;\mathrm{PM}}_{2.5\;}$ on air pollution-related mortality. The second objective of the study is to provide a better understanding of the sources contributing to ${\;\mathrm{PM}}_{2.5}$ and mortality by estimating the health impacts of coal consumption in China based on the multi-threshold effects of ${\;\mathrm{PM}}_{2.5}$ and ${\mathrm{SO}}_{2}$.

## 3. Methodology

### 3.1. Panel Regression Model

To depict the relationship between coal consumption and air pollution from different time periods and locations, a multiple panel regression model is estimated in Logarithmic form. The population-weighted exposure to ${\mathrm{PM}}_{2.5}$ is considered to be a dependent variable, while six factors (coal consumption, gasoline and diesel consumption, area of paved road per capita, temperature, precipitation, and humidity) are selected as main explanatory variables, since they are closely related to China air pollution and have frequently been used in the literature as discussed in the previous section.

The panel regression model that elucidates the relationship between fuel consumption and population-weighted exposure to ${\;\mathrm{PM}}_{2.5}$ is as follows:

(1) $$LnPM_{2.5it}\;=\beta_{0}+\beta_{1}LnCoal\_cons_{it}+\;\beta_{2}B08+\beta_{3}LnGasDie\_cons_{it}+\;\beta_{4}LnPavedRd_{it}+\beta_{5}LnTemp_{it}+\beta_{6}LnPrecp_{it}+\beta_{7}LnHumid_{it}+\;\varepsilon_{it}$$

for a balanced panel, where *i* and *t* denote province and time (year), $PM_{2.5it}$ is the regional population-weighted exposure to fine particulate matter, $Coal\_cons_{it}$ is the coal consumption by region, B08: a dummy variable that will be 1 if the data set is from 2004–2008, $GasDie\_cons_{it}$ is the summation of regional consumption of gasoline and diesel oil, $PavedRd_{it}$ is per capita area of paved road, $Temp_{it}$ is average temperature, $Precp_{it}$ is average precipitation and $Humid_{it}$ is average relative humidity, and $\varepsilon_{it}$ is the error term.

### 3.2. Panel Threshold Models

#### 3.2.1. Theoretical Model

This study employs Hansen’s panel threshold regression model in natural logarithmic form to further investigate the threshold effect of fine particulate matter on mortality rate.

The structure of the single panel threshold model is as follow:

(2) $$y_{it}=\mu_{i}+\beta_{1}^{\prime }x_{it}I\left(q_{it}\leq \gamma \right)+\beta_{2}^{\prime }x_{it}I\left(q_{it}>\gamma \right)+\epsilon_{it}\;$$

where the data are from a balanced panel, i and t denote indexes of the individual (1≤i≤N)  and the time (1≤t≤T), respectively; $y_{it}$ and the threshold variable, $q_{it}$, are scalars; $x_{it}$ is a k vector of explanation variables; I(∙) is an indicator function; $\mu_{i}$ is the fixed effect (or heterogeneity of individuals); and the error term, $\epsilon_{it}$, is assumed to be independent and identically distributed, $\epsilon_{it}\sim iid\left(0,\sigma^{2}\right)$. Equation (2) can be written as follows:

(2a) $$y_{it}=\mu_{i}+\beta^{\prime }x_{it}\left(\gamma \right)+\epsilon_{it}$$

where $\beta^{\prime }x_{it}\left(\gamma \right)=\{\begin{array}{c} \beta_{1}^{\prime }x_{it}I\left(q_{it}\leq \gamma \right)\\ \beta_{2}^{\prime }x_{it}I(q_{it}>\gamma ) \end{array}$

The data are separated into two regimes, whereby the threshold variable, $q_{it}$, is less than or greater than the threshold value, γ. The two regimes have different regression slopes, $\beta_{1}^{\prime }$ and $\beta_{2}^{\prime }$, respectively.

Hansen extended the panel threshold model with more than one threshold, where the threshold value, $\gamma_{1}$, is less than $\gamma_{2}$, as follows:

(2b) $$y_{it}=\mu_{i}+\beta_{1}^{\prime }x_{it}I\left(q_{it}\leq \gamma_{1}\right)+\beta_{2}^{\prime }x_{it}I\left(\gamma_{1}<q_{it}\leq \gamma_{2}\right)+\beta_{3}^{\prime }x_{it}I\left(q_{it}>\gamma_{2}\right)+e_{it})$$

For more specific details on the model and threshold test, refer to Hansen [26].

#### 3.2.2. Empirical Model

For choosing an appropriate threshold variable for specific mortality rate, we consider comparing the health impacts of different air pollutants on mortality. In addition to significant mortality effects of ${\mathrm{PM}}_{2.5}$ on both of cardiovascular mortality and respiratory mortality discussed, many previous papers have shown that ${\mathrm{SO}}_{2}$ has the highest degree of impact compared to ${\mathrm{PM}}_{2.5}$ and ${\mathrm{NO}}_{2}$ in terms of respiratory mortality [48,49]. Hence, we decided to choose ${\mathrm{PM}}_{2.5}$ for depicting the threshold effect on the cardiovascular mortality rate, and ${\mathrm{SO}}_{2}$ is chosen as the threshold variable for estimating the health effect regarding respiratory mortality. In each model, three air pollutants (${\mathrm{PM}}_{2.5}$, ${\mathrm{NO}}_{2}$, ${\mathrm{SO}}_{2}$) are selected as the main explanatory variables. The gross regional product (GRP) is also used, as a socioeconomic factor that is believed to influence the public health. The specific structure of the panel threshold models will be presented in this section.

We firstly apply the threshold test on ${\mathrm{PM}}_{2.5}$ for the mortality rate of cardiovascular to see whether any threshold relationship exists. The version with more regime-dependent coefficients enables estimating the health impacts of different air pollutants including ${\mathrm{PM}}_{2.5}$, ${\mathrm{SO}}_{2}$, and ${\mathrm{NO}}_{2}$, under each certain level of ${\mathrm{PM}}_{2.5}$ for individual thresholds. Suppose a triple threshold effect were found between cardiovascular mortality rate and ${\mathrm{PM}}_{2.5}$, the panel threshold model would be as follows:

(3) $$\begin{array}{ccc} LnMOT_{1it} & = & \mu_{i}\;+\;\left(\alpha_{1}LnPM_{2.5it-1}\;+\;\beta_{1}LnSO_{2it-1}\;+\;\theta_{1}.LnNO_{2it-1}\right)I\left(LnPM_{2.5it-1}\leq \gamma_{1}\right)\\ & & \;+\;(\alpha_{2}LnPM_{2.5it-1}\;+\;\beta_{2}LnSO_{2it-1}\\ & & \;+\;\theta_{2}.LnNO_{2it-1})I\left(\gamma_{1}<Ln{\mathrm{PM}}_{2.5\mathrm{it}-1}\leq \gamma_{2}\right)\;+\;(\alpha_{3}LnPM_{2.5it-1}\\ & & \;+\;\beta_{3}LnSO_{2it-1}\;+\;\theta_{3}.LnNO_{2it-1})I\left(\gamma_{2}<Ln{\mathrm{PM}}_{2.5\mathrm{it}-1}\leq \gamma_{3}\right)\\ & & \;+\;(\alpha_{4}LnPM_{2.5it-1}\;+\;\beta_{4}LnSO_{2it-1}\\ & & \;+\;\theta_{4}.LnNO_{2it-1})I\left(LnPM_{2.5it-1}>\gamma_{3}\right)\;+\;\varphi_{3}.LnGRP_{it-1}\;+\;\epsilon_{\mathrm{it}} \end{array}$$

Regarding respiratory mortality rate, we develop a panel threshold model to find ${\mathrm{PM}}_{2.5}$ health effects under specific thresholds of ${\mathrm{SO}}_{2}$ emission level, as follows (supposing we found a single threshold effect for ${\mathrm{SO}}_{2}$):

(4) $$\begin{array}{ccc} LnMOT_{2it} & = & \mu_{i}\;+\;\alpha_{1}LnPM_{2.5it-1}I\left(LnSO_{2it-1}\leq r\right)\;+\;\alpha_{2}LnPM_{2.5it-1}I\left(LnSO_{2it-1}>r\right)\\ & & \;+\;\theta_{1}.LnSO_{2it-1}\;+\;\theta_{2}.LnNO_{2it-1}\;+\;\theta_{3}.LnGRP_{it-1}\;+\;\varepsilon_{it} \end{array}$$

where *i* and *t* denote province and time (year), $MOT_{kit}\;$ is the cause-specific mortality rate, which is $MOT_{1it}\;$ for cardiovascular mortality rate and $MOT_{2it}$ for respiratory disease mortality rate, $\mu_{i}$ is the fixed effect (controlling for the heterogeneity of individual regions), $PM_{2.5it}$ is the population-weighted exposure to ${\mathrm{PM}}_{2.5}$, $SO_{2it}$ is the volume of regional $SO_{2}$ emission, $NO_{2it}$ is the average concentration of $NO_{2}$, GRP is the gross regional product which refers to the final products at market prices produced by all resident units in a province during a certain period of time, we make the calculation using 2005 as the base year, and $\varepsilon_{it},$ $\epsilon_{it}$ are the error terms. The right-hand-side variables interpret the lagged effects of the independent variables on cause-specific mortality. The estimated health effect threshold equation allows mortality rate to vary as it crosses three thresholds of ${\;\mathrm{PM}}_{2.5}$, as shown in the results in the later section.

This study focuses on estimating the health impacts of air pollution based on the threshold effect of ${\;\mathrm{PM}}_{2.5}$, thereby using the unit of ${\mathrm{PM}}_{2.5}\;$ as 10 $\mu g/m^{3}$ not be appropriate, since this may create some complicated cases for explanation. For example, increasing a 10 $\mu g/m^{3}$ unit could move ${\mathrm{PM}}_{2.5}$ level from a low threshold to higher thresholds, with different health impacts for each. Hence, we use the natural logarithmic form for the models and will interpret the percentage change in mortality rate by a 1% ${\mathrm{PM}}_{2.5}$ increase.

### 3.3. Data Set

In this study, we use the data of annual population-weighted ${\mathrm{PM}}_{2.5}$ exposure of 30 Chinese provinces and municipalities estimated by a team of US scientists and provided by Hsu [50]. The 30 Chinese provinces are Beijing, Tianjin, Hebei, Shanxi, Inner Mongolia, Liaoning, Jilin, Heilongjiang, Shanghai, Jiangsu, Zhejiang, Anhui, Fujian, Jiangxi, Shandong, Henan, Hubei, Hunan, Guangdong, Guangxi, Hainan, Sichuan, Guizhou, Yunnan, Tibet, Shaanxi, Gansu, Qinghai, Ningxia, and Xinjiang. Population-weighted ${\mathrm{PM}}_{2.5}$ exposure for a specific province is calculated by multiplying the satellite-estimated ${\mathrm{PM}}_{2.5}$ concentration for each grid cell by the percentage of province population that lives within that grid cell and producing an average for all the grid cells within a province: PW − ${\mathrm{PM}}_{2.5}$ = $\sum_{i=1}^{n}({\mathrm{PM}}_{i}*\frac{P_{i}\;}{\sum_{i=1}^{n}P_{i}}\;)$, where ${\mathrm{PM}}_{i}\;$ is defined as the $i^{th}$ pixel value of satellite ${\mathrm{PM}}_{2.5}$ concentration, $P_{i}$ is the population density of the $i^{th}$ grid cell of a certain province, which is divided by n grid cells [51]. As can be seen from the formula, that PW-${\mathrm{PM}}_{2.5}$ implies that the exposure to ${\mathrm{PM}}_{2.5}\;$ in highly populated areas is greater than that in regions with sparse density. This indicator is more telling of actual exposure to ${\mathrm{PM}}_{2.5}$ and more in line with actual pollution situation compared to per capita ${\mathrm{PM}}_{2.5}$ concentration [51]. Hence, it would be appropriate for investigating the health consequences of poor air quality.

Data on other air pollutants (i.e., ${\mathrm{SO}}_{2}\;$ emission, ${\mathrm{NO}}_{2}$ concentration), energy consumption (i.e., consumption of coal; total consumption of gasoline and diesel oil), meteorological conditions (i.e., average temperature, relative humidity and precipitation), per capita area of paved road, and socioeconomic factors, i.e., gross regional product (GRP), were retrieved from the National Bureau of Statistics of China [52]. Table 1 displays descriptive statistics on these variables. The mean annual ${\mathrm{PM}}_{2.5}$ level of China is 27.26 $\mu g/m^{3}$ and most provinces and municipalities exceed the ${\mathrm{PM}}_{2.5}$ standard level proposed by WHO, which is set at 10 $\mu g/m^{3}$.

The mortality estimates for specific causes including cardiovascular mortality and respiratory mortality are provided by the Institute for Health Metrics and Evaluation, University of Washington, Seattle, USA under the Global of Burden Disease Study 2013 [53]. Due to the lack of frequent yearly data of mortality estimates, we convert the data in 5-year intervals provided into annual datasets using the Geometric Power Series simulation method. Once annual cause-specific mortality estimates have been simulated, the mortality rate of each province is calculated by dividing mortality estimates by the average population of the province, and then multiplying by 100,000. Provincial average population data is released by the National Bureau Statistical of China. Table 2 displays descriptive statistics for variables those are used for estimating the threshold effects of ${\mathrm{PM}}_{2.5}$ on mortality rate.

**Figure 1:**
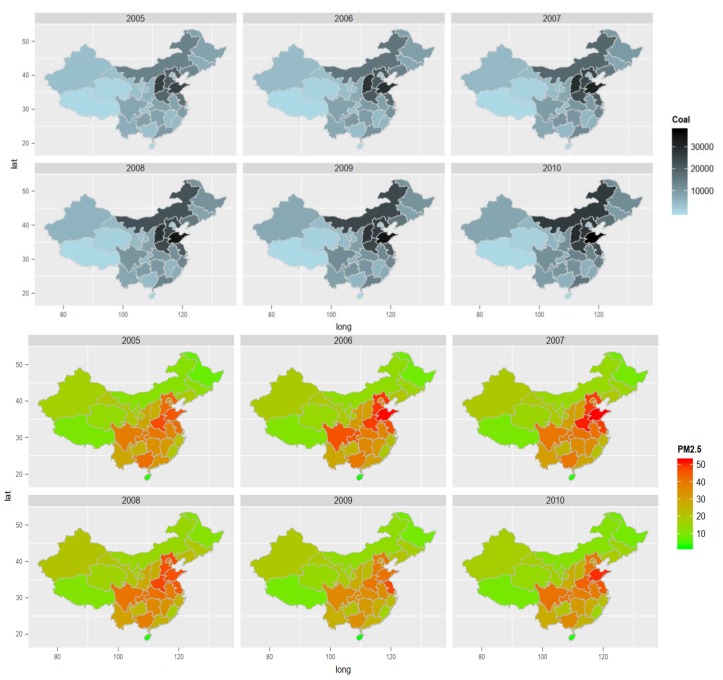
Mapping annual coal consumption and surface distribution of population-weighted exposure to ${\mathrm{PM}}_{2.5}$ concentration in China for the years 2005 to 2010.

Using the ggplot2 package in R-studio, we map the annual coal consumption and population-weighted exposure to ${\mathrm{PM}}_{2.5}$ concentration data to provide a visualization of spatial distribution and temporal changes for a 5-year time trend. In Figure 1, we can partially observe the correlation between consumption of coal and fine particle pollution through the similarities in the intensity of the color mapped.

## 4. Results

### 4.1. Estimating the Environmental Impacts of Fossil Fuel Consumption

To deal with common panel data problems such as heteroscedasticity, autocorrelation, or cross-correlation in cross-sectional units at the same point in time, we run pooled ordinary least squares (OLS), fixed effects (FE) model, and random effects (RE) model. The results of F test, LM test indicate that fixed effects model and random effects model are both better than the pooled model. Since the balanced panel data has 29 cross-sections and only 7 years, the sum squares of residuals decreased tremendously, leading to an increase of adjusted $R^{2}$ in the fixed effects model. Therefore, technically, the adjusted $R^{2}$ of FE model is always higher than in RE model assuming the same specification. We depend on the Hausman test, and its results show that the random effects model is the most appropriate model for our data. The estimated parameters for the panel multiple regression are displayed in Table 3.

At a significance level of 1%, we find that a 1% coal consumption increase leads to a 0.23% increase in population-weighted exposure to ${\;\mathrm{PM}}_{2.5}$. The volume of gasoline and diesel oil consumption has been found with a positive effect on ${\mathrm{PM}}_{2.5}$ with a 0.065% increase in ${\mathrm{PM}}_{2.5}$ concentration if there is a 1% consumption of gasoline and diesel increase. We also find that estimated coefficient results of three meteorological variables are all significant at 1% and 5%, showing us the strong sensitivity of population-weighted ${\mathrm{PM}}_{2.5}$ exposure to climate change. Temperature and relative humidity are positively correlated with ${\mathrm{PM}}_{2.5}$, while average precipitation and ${\mathrm{PM}}_{2.5}$ have a negative correlation. These estimated results have similar trends with the results in another research that using an 11-year observational record over the contiguous US [37].

In addition, the estimated coefficient of dummy variable B08 indicates that the difference in population-weighted ${\mathrm{PM}}_{2.5}$ exposure before and after 2008, year the Olympics were held, is statistically significant at 1% level and the concentration of ${\mathrm{PM}}_{2.5}$ after 2008 is lower than in 2004–2008 by about 14.5%. This means the achievements from China’s efforts in providing a better air quality during the 2008 Olympic Games are significant.

### 4.2. Estimating Multiple Threshold Effects of $PM_{2.5}$ on Mortality

#### 4.2.1. Testing for Multiple Thresholds

A logarithmic version of Hansen’s threshold model is estimated using a panel data approach, as a panel threshold model (PTM). Table 4 shows the results of the threshold effect tests.

For the ${\mathrm{PM}}_{2.5}$–cardiovascular mortality (${\mathrm{MOT}}_{1}$) relationship, the single and triple effects are all significant at a 1% level. We choose the triple threshold effect for further estimation to see the health impacts of ${\mathrm{PM}}_{2.5}$’s thresholds more thoroughly. These three estimated thresholds (Table 5) are 17.67 μg/$m^{3}$, 21.62 μg/$m^{3}$ and 34.27 μg/$m^{3}$ with narrow confidence intervals.

In terms of respiratory mortality (${\mathrm{MOT}}_{2}$), ${\mathrm{SO}}_{2}$ emission is selected as the threshold variable instead. Table 4 shows that the double effect is not significant, while the test results of the single and triple effects are significant at 1% and 5%, respectively. Hence, we choose a single effect based on the lowest P-value. The estimated threshold of regional ${\mathrm{SO}}_{2}$ mission (Table 6) is 80.13 tons/year.

**Figure 2:**
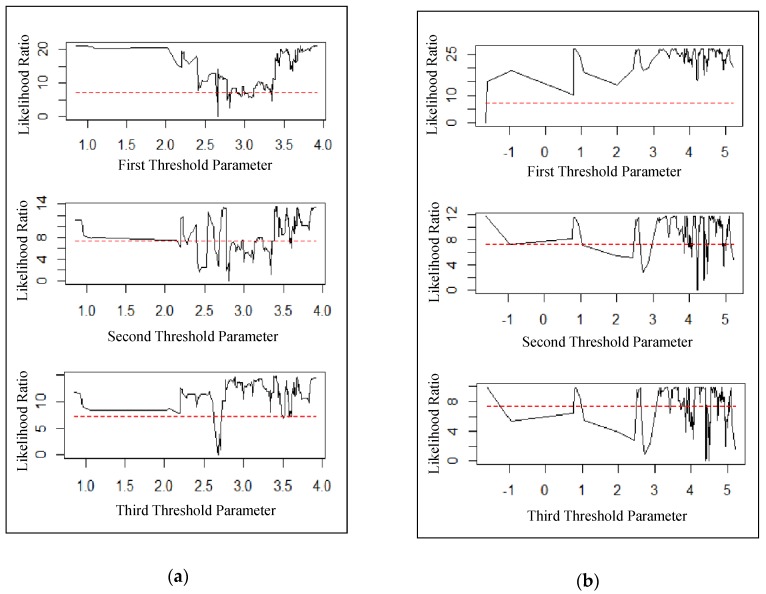
Confidence interval construction in (**a**) ${\mathrm{PM}}_{2.5}$ thresholds and (**b**)${\;\mathrm{SO}}_{2}$ thresholds. Note: The dashed line denotes the critical value (7.35) at the 95% confidence level.

As the best way to form confidence intervals for threshold is to form “no-rejection region” using the likelihood-ratio (LR) statistic for tests on threshold estimates [26], we plot the LR statistic (Figure 2) to display the threshold confidence intervals.

In Figure 2a, the LR statistic of the third threshold mostly exceeds α quantile (7.35), while LR values of the first and the second threshold do not, which means that there exists a triple threshold effect in the relationship between ${\mathrm{PM}}_{2.5}$ and cardiovascular mortality rate. Regarding association between ${\mathrm{SO}}_{2}$ pollution and respiratory mortality rate, Figure 2b shows that only a single threshold effect is significant.

#### 4.2.2. Estimated Effects of ${\mathrm{PM}}_{2.5}$ on Cause-Specific Mortality Rate

As in Table 5, the estimated coefficients of the air pollutants are different compared to each other and to itself under specific threshold levels of the annual population-weighted ${\mathrm{PM}}_{2.5}$ exposure. We find that when ${\mathrm{PM}}_{2.5}$ is between 17.67 μg/$m^{3}$ and 21.62 μg/$m^{3}$, a 1% ${\mathrm{PM}}_{2.5}$ increase leads to a 0.80% increase in mortality rate of cardiovascular disease. When ${\mathrm{PM}}_{2.5}$ is between 21.62 μg/$m^{3}$ and 34.27 μg/$m^{3}$, a 1% ${\mathrm{PM}}_{2.5}$ increase results in a 0.26% increase in the mortality rate.

The regression estimates of ${\mathrm{NO}}_{2}$ and ${\mathrm{SO}}_{2}$ show that when the annual ${\mathrm{PM}}_{2.5}$ exposure is lower than 17.67 μg/$m^{3}$, a 1% increase in ${\mathrm{NO}}_{2}\;$ concentration leads to a 0.19% increase in cardiovascular mortality rate, while the health impact of ${\mathrm{SO}}_{2}$ is not significant. When ${\mathrm{PM}}_{2.5}$ is between 21.62 μg/$m^{3}$ and 34.27 μg/$m^{3}$, a 1% increase in ${\mathrm{SO}}_{2}$ emission causes 0.05% cardiovascular mortality rate increase, and when ${\mathrm{PM}}_{2.5}$ is higher than 34.27 μg/$m^{3}$, this impact is increased by 0.17%. However, we get negative signs for the estimated coefficients of ${\mathrm{SO}}_{2}$ and ${\mathrm{NO}}_{2}$ under the second and the third regimes.

In terms of respiratory mortality rate, Table 6 displays the panel threshold model outcomes, indicating that the impact of ${\mathrm{PM}}_{2.5}$ depends on the initial ${\mathrm{SO}}_{2}$ emission. When the average regional ${\mathrm{SO}}_{2}$ emission is lower than 80.13 tons/year, a 1% increase in ${\mathrm{PM}}_{2.5}$ leads to an increment of 0.17% in mortality rate. When ${\mathrm{SO}}_{2}$ is higher 80.13 tons/year, a 1% increase in ${\mathrm{PM}}_{2.5}$ increases the respiratory mortality rate by 0.25%. The ${\mathrm{NO}}_{2}$ emission is also found to be associated with human health where a 1% increase in ${\mathrm{NO}}_{2}$ causes respiratory mortality increased by 0.17%.

We find a significant positive impact of the economic developing where a 1% increase in GRP increases the cardiovascular mortality by 0.04%. However, we find a negative relationship between GRP and respiratory mortality rate.

#### 4.2.3. Two-stage Econometric Approach for Health Effects of Coal Consumption

Equation (1) allows us to estimate the change in ${\mathrm{PM}}_{2.5}$ given by the change in coal consumption. We then combine this with Equation (3) and Equation (4), which found the threshold effects of ${\;\mathrm{PM}}_{2.5}$ on cause-specific mortality, to calculate the percentage change in mortality corresponded to a 1% change in coal consumption. We do this by multiplying the estimated coefficient of coal consumption and the values of regression estimates of ${\mathrm{PM}}_{2.5}$ together. Table 7 displays the results.

The final outcome of the two-stage approach indicate that cardiovascular mortality rate increases by 0.188% when coal consumption increases by 1% in regions where the population-weighted ${\mathrm{PM}}_{2.5}$ exposure is between 17.67 μg/$m^{3}$ and 21.62 μg/$m^{3}$, and that it increases by 0.06% for a 1% increase in coal consumption when ${\mathrm{PM}}_{2.5}$ is between 21.62 μg/$m^{3}$ and 34.27 μg/$m^{3}$. In terms of respiratory mortality, when the regional ${\mathrm{SO}}_{2}$ emission is less than 80.13 tons/year, under the health impacts of ${\mathrm{PM}}_{2.5}$, a 1% coal consumption increase leads to a 0.04% increase in mortality rate. When ${\mathrm{SO}}_{2}$ emission is greater than 80.13 tons per year, a 1% increase in coal consumption leads to a 0.058% increase in respiratory mortality due to exposure to ${\mathrm{PM}}_{2.5}$.

## 5. Discussion

Regarding the trend of the estimated coefficient of ${\mathrm{PM}}_{2.5}$ for different thresholds of ${\mathrm{PM}}_{2.5}$, the health effect at the second regime has the highest degree of impact on cardiovascular mortality rate. However, the effect became less serious for the next regime of exposure to ${\mathrm{PM}}_{2.5}$, afterward. This could be explained by human awareness of air pollution leading people to protect themselves from bad air quality. When the level of ${\mathrm{PM}}_{2.5}$ is too high, it could be visually identified by the citizens or be notified officially by a red alert with respect to air pollution; thus, people become more aware of the dangerous characteristics of pollution levels and are more active in protecting their own health from the polluted air. Mortality rate per 1% increment of ${\mathrm{PM}}_{2.5}$ tended to decrease at higher concentrations in accordance with prior findings from Pope, et al. [54] and Pope III, Burnett, Turner, Cohen, Krewski, Jerrett, Gapstur and Thun [7], which showed the adjusted relative risk of cardiovascular plotted over estimated daily dose of ${\mathrm{PM}}_{2.5}$ and found that the exposure-response curve becomes flatter at higher degree of ${\mathrm{PM}}_{2.5}$. According to Chen, et al. [55], it was inferred that possibly susceptible individuals may have died before the ${\mathrm{PM}}_{2.5}\;$ concentration had reached higher levels. Thus, due to the severe health damage at the medium regimes of ${\mathrm{PM}}_{2.5}$ threshold, people should pay more attention to these levels because the human body suffers gradually from exposure to air pollutants from low to high concentrations.

${\mathrm{SO}}_{2}$ is formed when fossil fuels containing sulfur, such as coal or crude oil, are burned, and this air pollutant is known to be one of the major causes of respiratory mortality. Since different levels of ${\mathrm{SO}}_{2}$ emission lead to difference in ${\mathrm{PM}}_{2.5}$ concentrations, the health impacts of ${\mathrm{PM}}_{2.5}$ are not below the emission thresholds of sulfur dioxide to the same degree. Particularly, the mortality effect of ${\mathrm{PM}}_{2.5}$ becomes more severe at higher levels of ${\mathrm{SO}}_{2}$ emission. Compared to findings from previous research, where the acute effect of ${\mathrm{SO}}_{2}$ on respiratory disease mortalities was even higher than that from ${\mathrm{PM}}_{2.5}$ or ${\mathrm{NO}}_{2}$ [48,49], this study contributes to the existing literature as evidence of adverse effects of ${\mathrm{SO}}_{2}$ on respiratory mortality, no matter whether it is studied separately or through ${\mathrm{PM}}_{2.5}$.

As mentioned, this study estimates the percentage of change in mortality given a percentage change in ${\mathrm{PM}}_{2.5}$, to appropriately depict the exposure-response relation with multiple threshold effects. However, to better compare our findings to previous studies, we convert the outcomes to comparable values by dividing the estimates in the fourth column of Table 7 by average ${\mathrm{PM}}_{2.5}$, and multiplied by 1000. In this way, converted estimates of health effect showed the percentage change in cause-specific mortality caused by a 10 μg/$m^{3}$ increase in ${\;\mathrm{PM}}_{2.5}$. Table 8 presents the results in comparison with previous studies.

As can be seen, the health effects for the ranges in which population-weighted ${\mathrm{PM}}_{2.5}$ exposure falls are larger than the short-term effects and smaller than health effects from long-term exposure in China. For instance, for each 10 μg/$m^{3}$ increase in ${\;\mathrm{PM}}_{2.5}$, the short-term (daily) exposure caused a 0.41% and 0.95% increase of cardiovascular and respiratory mortality, respectively [19], a 1-year lagged effect of ${\mathrm{PM}}_{2.5}$ exposure that was conducted in this study leads to a 9.63–30.18% increment in cardiovascular mortality rate and about 6.45–9.35% in mortality rate for respiratory diseases (Table 8), while the long-term exposure to particulate matter in 10-year follow up studies has reported that it caused an increase of 55% in cardiovascular mortality and 67% in respiratory mortality in Shenyang, China [39,40], or an increase of 31% in ischemic cardiovascular mortality in Canada [24].

## 6. Conclusions

### 6.1. Contribution of the Study

The effect of ambient air pollution on human health has been extensively studied over the past five decades. Among the research directions that are motivated, providing convincing evidence of nonlinear exposure-response relationship with specific ${\mathrm{PM}}_{2.5}$ thresholds could provide useful information for efforts to protect public health from the impacts of poor air quality. However, the threshold effects of population-weighted ${\mathrm{PM}}_{2.5}$ exposure on cause-specific mortality in China have not been estimated. In this study, we investigate this issue.

The empirical results indicate that air pollution will influence mortality of cardiovascular and respiratory diseases through ${\mathrm{PM}}_{2.5}$ levels. We find that the effects depend on the ranges in which air pollutant falls, with critical levels at 17.67 μg/$m^{3}$, 21.62 μg/$m^{3}$, and 34.27 μg/$m^{3}$ for ${\mathrm{PM}}_{2.5}$ concentration, at 80.13 tons/year for ${\mathrm{SO}}_{2}$ emission. In particular, between 17.67 μg/$m^{3}$ and 21.62 μg/$m^{3}$, we find that a 1% increase in ${\mathrm{PM}}_{2.5}$ causes a 0.8% increase in cardiovascular mortality rate. When ${\mathrm{SO}}_{2}$ is above 80.13 tons/year, a 1% increase in ${\mathrm{PM}}_{2.5}$ causes a 0.25% increase in respiratory mortality rate. We also estimate the effect of coal consumption, where a 1% increase in coal consumption causes a 0.23% increase in ${\mathrm{PM}}_{2.5}$ concentration.

Our two-stage econometric approach allows us to examine the health effects of coal consumption through multiple threshold effects of ${\;\mathrm{PM}}_{2.5}$. The result shows that when exposure to ${\mathrm{PM}}_{2.5}$ is between 17.67 μg/$m^{3}$ and 21.62 μg/$m^{3}$, a 1% increase in coal consumption leads to a 0.188% increase in cardiovascular mortality rate. In terms of mortality, when ${\mathrm{SO}}_{2}$ emission is greater than 80.13 tons/year, under the health impacts of ${\mathrm{PM}}_{2.5}$, a 1% increase in coal consumption leads to a 0.06% increase in respiratory mortality rate.

The findings indicate that the consumption of fossil fuel energy is a major cause of particulate air pollution and lead to further adverse cardiovascular and respiratory health impacts.

### 6.2. Implications of the Study

Compared to the standards of WHO Air Quality Guideline and the National Ambient Air Quality Standard of China (GB 3095–2012), 17.67 μg/$m^{3}$ is close to the lowest annual ${\mathrm{PM}}_{2.5}$ level of the China standard (grade I), and the WHO interim target-3 (IT-3) is 15 μg/$m^{3}$. In addition, the third estimated ${\mathrm{PM}}_{2.5}$ threshold (34.27 μg/$m^{3}$) seems to be close to the highest standard levels, such as IT-1 of the WHO standard and limit value of grade II in the GB 3095–2012 standard (35 μg/$m^{3}$). The findings of threshold effect provide an intuitive metric of ${\mathrm{PM}}_{2.5}$ standards to reinforce or improve current air quality standards, especially for countries with high level of air pollutants. The estimates of health effects under the population-weighted ${\;\mathrm{PM}}_{2.5}$’s thresholds could be considered to set more specific emission limits or punishment levels for controlling polluting activities in China and other developing countries. It is also important to impose air quality control strategies that consider population density of area since “population-weighted” exposure to ${\mathrm{PM}}_{2.5}$ is found significantly associated with mortality rate.

In addition, owing to the significant association between fossil fuel consumption and mortality rates, it is necessary to reduce our dependence on this polluting energy sources. The improvement achieved in reducing air pollution during and after 2008 is strong evidence that supports for optimistic future of China’s air if the government continues durable clean air plans.

### 6.3. Limitations of the Study

Despite the large-scale study area and the remarkable contribution to finding out the thresholds of ${\;\mathrm{PM}}_{2.5}$, the database approach is a constraint with some limitations. The first limitation concerns the data of regional cause-specific mortality. Due to the lack of available annual regional cause-specific mortality, the annual data of mortality in this study are simulated from the GDB 5-year-interval mortality data. This simulation may cause unusual changes in real mortality rate to be ignored, leading to unconvincing estimation outcomes. The second limitation is rooted in the difference in units of measurement for the air pollutants: population-weighted ${\mathrm{PM}}_{2.5}$ exposure (μg/$m^{3}$), ${\mathrm{SO}}_{2}$ emission (10,000 tons) and ${\mathrm{NO}}_{2}$ concentration (μg/$m^{3}$). Thus, the health effects of specific air pollutants are not really logical to interpret and draw comparisons between them as well. Additionally, only population-weighted ${\mathrm{PM}}_{2.5}$ concentration data without considering human activities with time spent and breathing rates could not be considered as “exposure to ${\mathrm{PM}}_{2.5}$” to accurately estimate its health impacts. This is the third limitation.

### 6.4. Recommendations for Further Research

As we need limitations to inspire better thinking, we firstly recommend that further studies use a more appropriate database, such as higher frequency data or data better representing the actual exposure to ${\mathrm{PM}}_{2.5}$, to achieve higher-quality estimation results. Second, health impacts caused by acute ${\mathrm{PM}}_{2.5}$ exposure are not reflected by mortality rate alone; estimation of reduction in life expectancy, or increases in morbidity may be preferable. Finally, the non-significance of the ${\mathrm{PM}}_{2.5}$ lowest threshold could be considered a safe threshold of ${\mathrm{PM}}_{2.5}$ for cardiovascular mortality. Future research is also encouraged in the direction of identifying a safe threshold of exposure to ${\mathrm{PM}}_{2.5}$ on cause-specific mortality and morbidity for contributing more evidence supports environmental science even though they may be different from this study.

## Figures and Tables

**Table 1:** Descriptive statistics for variables in panel regression model.

Variables	Description	Mean	Median	Max	Min	Std. Dev.
${\mathrm{PM}}_{2.5}$	${\mathrm{PM}}_{2.5}$ concentration $(\mu g/m^{3}$)	27.26	26.96	51.91	2.17	11.71
Coal_cons	coal consumption (10,000 tons)	10,520.76	8559.73	37,327.89	332.23	7897.92
GasDie_cons	gasoline-diesel consumption (10,000 tons)	682.03	568.77	2754.68	40.74	496.26
Paved_Rd	per capita area of paved road (sq.m)	11.41	11.19	22.23	4.04	3.31
Temp	average temperature (℃)	14.47	15.1	25.4	4.5	5.07
Humid	relative humidity (%)	64.24	66	83	44	9.41
Precp	precipitation (mm)	867.63	765.6	2628.2	74.9	503.46
Observations	203					

**Table 2:** Descriptive statistics for variables in panel threshold regression model.

Variables	Description	Mean	Median	Max	Min	Std. Dev.
${\mathrm{MOT}}_{1}$	cardiovascular mortality rate (deaths per 100,000 persons)	238.84	239.02	355.85	152.94	49.04
${\mathrm{MOT}}_{2}$	respiratory mortality rate (deaths per 100,000 persons)	114.39	104.42	226.05	55.73	43.25
${\mathrm{PM}}_{2.5}$	${\mathrm{PM}}_{2.5}$ concentration ($\mu g/m^{3}$)	26.69	26.72	51.94	2.17	11.92
${\mathrm{NO}}_{2}$	${\mathrm{NO}}_{2}$ concentration ($\mu g/m^{3}$)	40.86	41.30	73.00	11.90	13.69
${\mathrm{SO}}_{2}$	${\mathrm{SO}}_{2}$ emission (10,000 tons)	76.30	63.35	200.30	0.10	48.16
GRP	gross regional product (100 million yuan)	8279.15	6438.74	35,696.71	229.04	7139.56
Observations	210					

**Table 3:** Panel regression estimation results of impact of burning coal effects on ${\mathrm{PM}}_{2.5}$.

Variables	Pooled OLS	FE Model	RE Model
	Coefficients		
Constant	−1.345 (1.228)	−1.771 ** (0.829)	−1.923 ** (0.755)
LnCoal_cons	0.404 *** (0.0544)	0.196 *** (0.056)	0.233 *** (0.051)
B08	0.126 * (0.0704)	0.142 *** (0.0164)	0.145 *** (0.016)
LnGasDie_cons	0.0168 (0.069)	0.076 ** (0.038)	0.0650 * (0.037)
LnPaved_Rd	−0.158 (0.106)	−0.046 (0.038)	−0.056 (0.037)
LnTemp	0.689 *** (0.106)	0.233 ** (0.110)	0.241 ** (0.095)
LnHumid	0.244 (0.365)	0.604 *** (0.159)	0.581 *** (0.149)
LnPrec	−0.260 ** (0.101)	−0.059 ** (0.027)	−0.061 ** (0.027)
Adj R^2^	0.481	0.984	0.376
F test (Pooled vs. Fixed)		216.96 ***	
LM test (Pooled vs. Random)			540.24 ***
Hausman Test (Random vs. Fixed)			6.04

Note: Standard errors in parentheses. *, ** and ***, respectively, denote significance at the 10%, 5% and 1% levels.

**Table 4:** Tests for threshold effects.

Threshold	${\mathrm{PM}}_{2.5}\;-\;{\mathrm{MOT}}_{1}\;\mathrm{Relationship}$	${\mathrm{SO}}_{2}\;-\;{\mathrm{MOT}}_{2}\;\mathrm{Relationship}$
Test for single threshold		
$F_{1}$	210.329	51.314
*p*-value	0.000	0.010
Critical values (10%, 5%, 1%)	29.720, 37.778, 49.981	27.013, 36.152, 45.191
Test for double threshold		
$F_{2}$	24.799	17.189
*p*-value	0.080	0.250
Critical values (10%, 5%, 1%)	23.274, 28.334, 34.176	23.340, 28.697, 36.832
Test for triple threshold		
$F_{3}$	142.326	21.743
*p*-value	0.000	0.013
Critical values (10%, 5%, 1%)	20.952, 27.461, 41.740	13.272, 16.893, 22.610

**Table 5:** Estimation results of ${\mathrm{PM}}_{2.5}$ effects on cardiovascular mortality.

Threshold Estimates	Threshold	Estimates	95% Confidence	$e^{\left(\mathrm{threshold}\right)}$
	$\gamma_{1}$	2.872	[2.717, 2.872]	17.67
	$\gamma_{2}$	3.073	[3.074, 3.074]	21.62
	$\gamma_{3}$	3.534	[2.872, 3.610]	34.27
	Variable	Coefficient	Regime-dependent	
			OLS S.E.	White S.E.
${\mathrm{PM}}_{2.5}$≤ 17.67	${\mathrm{LnPM}}_{2.5}$	0.031	0.043	0.060
	${\mathrm{LnSO}}_{2}$	−0.003	0.020	0.013
	${\mathrm{LnNO}}_{2}$	0.196 **	0.073	0.090
21.62 ≥ ${\mathrm{PM}}_{2.5}\;$> 17.67	${\mathrm{LnPM}}_{2.5}$	0.806 ***	0.245	0.198
	${\mathrm{LnSO}}_{2}$	−0.270 ***	0.078	0.064
	${\mathrm{LnNO}}_{2}$	−0.159	0.142	0.118
34.27 ≥ ${\mathrm{PM}}_{2.5}\;$> 21.62	${\mathrm{LnPM}}_{2.5}$	0.257 ***	0.059	0.054
	${\mathrm{LnSO}}_{2}$	0.054 *	0.025	0.028
	${\mathrm{LnNO}}_{2}$	−0.162 ***	0.045	0.042
${\mathrm{PM}}_{2.5}\;$> 34.27	${\mathrm{LnPM}}_{2.5}$	−0.003	0.070	0.062
	${\mathrm{LnSO}}_{2}$	0.172 ***	0.028	0.020
	${\mathrm{LnNO}}_{2}$	−0.034	0.043	0.038
	Variable	Coefficient	Regime-independent	
			OLS S.E.	White S.E.
	LnGRP	0.0378 **	0.016	0.017

Note: White S.E. denotes heteroscedasticity-consistent standard errors. *, ** and ***, respectively, denote significance at the 10%, 5%, and 1% levels using the T-critical value.

**Table 6:** Estimation results of ${\mathrm{PM}}_{2.5}$ effects on respiratory mortality.

Threshold Estimates	Threshold	Estimates	95% Confidence	$e^{\left(\mathrm{threshold}\right)}$
	r	4.3836	[4.3549, 4.3993]	80.13
Variable	Coefficient	Regime-independent		
		OLS S.E.	White S.E.	*t*-statistic
${\mathrm{LnPM}}_{2.5}$ (${\mathrm{SO}}_{2}$ ≤ 80.13)	0.172 ***	0.028	0.043	4.050
${\mathrm{LnPM}}_{2.5}\;({\mathrm{SO}}_{2}>\;$80.13)	0.250 ***	0.029	0.047	5.352
Ln ${\mathrm{SO}}_{2}$	−0.032	0.019	0.035	−0.904
Ln ${\mathrm{NO}}_{2}$	0.176 ***	0.041	0.043	4.052
LnGRP	−0.188 ***	0.019	0.025	−7.585

Note: White S.E. denotes heteroscedasticity-consistent standard errors. ** and ***, respectively, denote significance at the 5%, and 1% levels using the T-critical value.

**Table 7:** Estimation results on the health impacts of coal consumption in air pollution.

Result of Stage 1 (Panel Data Model)	Result of Stage 2 (Panel Threshold Model)			Result of Two-Stage Approach
Estimate effect of coal consumption on ${\mathrm{PM}}_{2.5}$	Estimate effect of ${\mathrm{PM}}_{2.5}$ on cause-specific mortality			Estimate health effect of coal consumption
0.233 ***		Estimated threshold regimes	Coefficient	Coefficient
	Cardiovascular mortality	21.62 ≥ ${\mathrm{PM}}_{2.5}\;$> 17.67$\;\left(\mu g/m^{3}\right)$	0.806 ***	0.188
		34.27 ≥ ${\mathrm{PM}}_{2.5}\;$> 21.62$\;\left(\mu g/m^{3}\right)$	0.257 ***	0.060
	Respiratory mortality	${\mathrm{SO}}_{2}$≤ 80.13 (tons)	0.172 ***	0.040
		${\mathrm{SO}}_{2}>\;$80.13 (tons)	0.250 ***	0.058

**Table 8:** The percentage change in cause-specific mortality associated with a 10 $\mu g/m^{3}$ increase in ${\mathrm{PM}}_{2.5}.$

Study Approach	Regions [Author]	Pollutant	Methodology (Time Period)	Health Outcomes	Estimated Coef.
Short-term Studies	Shanghai, China [19]	${\mathrm{PM}}_{2.5}$	Time-series	Cardiovascular mortality	0.41 [0.00, 0.82]
				Respiratory mortality	0.95 [0.17, 1.73]
	Shenyang, China [18]	${\mathrm{PM}}_{2.5}$	Time-stratified case-crossover	Cardiovascular mortality	0.53 [0.09, 0.97]
				Respiratory mortality	0.97 [0.01, 1.94]
	Quangzhou, China [17]	${\mathrm{PM}}_{2.5}$	Time-stratified case-crossover	Cardiovascular mortality	1.22 [0.63, 1.68]
				Respiratory mortality	0.97 [0.16, 1.79]
This study	China	${\mathrm{PM}}_{2.5}$	Panel Threshold Model	Cardiovascular mortality	30.18 (21.62 ≥ ${\mathrm{PM}}_{2.5}\;$> 17.67)
					9.63 (34.27 ≥ ${\mathrm{PM}}_{2.5}\;$> 21.62)
				Respiratory mortality	6.45 (${\mathrm{SO}}_{2}$ ≤ 80.13)
					9.35 (${\mathrm{SO}}_{2}$ > 80.13)
Long-term Studies	Shenyang, China [39]	${\mathrm{PM}}_{10}$	Retrospective cohort study (1998–2009)	Cardiovascular mortality	55 [51, 60]
	Shenyang, China [40]	${\mathrm{PM}}_{10}$	Retrospective cohort (1998–2009)	Respiratory mortality	67 [60, 74]
	US metropolitan areas [56]	${\mathrm{PM}}_{2.5}$	Cohort study (1979–1983)	Cardiopulmonary mortality	6 [2, 10]
	Netherlands [57]	${\mathrm{PM}}_{2.5}$	Cohort study (1987–1996)	Respiratory mortality	7 [−25, 52]
	US metropolitan areas [56]	${\mathrm{PM}}_{2.5}$	Cohort study (1979–1983)	Cardiopulmonary mortality	6 [2, 10]
	Canada [24]	${\mathrm{PM}}_{2.5}$	Cohort study (1991–2001)	Cardiovascular mortality	31 [27, 35]

Note: [] refers to 95% confidence interval.
